# IscR Regulation of Type 3 Fimbriae Expression in *Klebsiella pneumoniae* CG43

**DOI:** 10.3389/fmicb.2017.01984

**Published:** 2017-10-16

**Authors:** Tien-Huang Lin, Cheng-Yin Tseng, Yi-Chyi Lai, Chien-Chen Wu, Chun-Fa Huang, Ching-Ting Lin

**Affiliations:** ^1^Division of Urology, Taichung Tzu Chi General Hospital, The Buddhist Tzu Chi Medical Foundation, Taichung, Taiwan; ^2^School of Post-Baccalaureate Chinese Medicine, Tzu Chi University, Hualien, Taiwan; ^3^Graduate Institute of Chinese Medicine, China Medical University, Taichung, Taiwan; ^4^Section of Infectious Disease, Hsinchu Mackay Memorial Hospital, Hsinchu, Taiwan; ^5^Department of Microbiology and Immunology, Chung-Shan Medical University, Taichung, Taiwan; ^6^School of Chinese Medicine, China Medical University, Taichung, Taiwan

**Keywords:** *Klebsiella pneumoniae*, IscR, type 3 fimbriae, biofilm formation, MrkHI

## Abstract

In *Klebsiella pneumoniae*, we have previously shown that IscR, an Fe–S cluster-containing transcriptional factor, plays a dual role in controlling capsular polysaccharide biosynthesis and iron-acquisition systems by switching between its holo and apo forms. In this study, the effect of IscR on type 3 fimbriae expression and biofilm formation was investigated. We found that production of the major subunit of type 3 fimbriae, MrkA, was increased in the Δ*iscR* and *iscR*_3CA_ strains, a strain expressing a mutant IscR that mimics apo-IscR, at both the translational and transcriptional levels. Based on the fact that type 3 fimbriae expression is the major factor affecting biofilm formation, increased biofilm formation was also found in Δ*iscR* or *iscR*_3CA_, suggesting that holo-IscR represses biofilm formation. However, the repression of type 3 fimbriae expression by IscR is indirect. To further understand the regulatory mechanism of IscR, the effect of IscR on the expression of *mrkHIJ*, which encodes cyclic di-GMP (c-di-GMP)-related regulatory proteins that control type 3 fimbriae expression, was studied. We found that holo-IscR could directly repress *mrkHI* transcription, indicating that MrkHI is required for IscR regulation of type 3 fimbriae expression. Finally, deletion of *iscR* attenuated *K. pneumoniae* virulence in a peritonitis model of mouse infection, while the absence of the [2Fe–2S] cluster of IscR had no effect on *K. pneumoniae* virulence during infection. Taken together, our results demonstrate the underlying mechanism of the [2Fe–2S] cluster of IscR in controlling type 3 fimbriae expression and its effect on *K. pneumoniae* pathogenesis.

## Introduction

Iron is essential to most bacteria for growth and reproduction. It plays a key role as a cofactor in the electron transport chain and for various enzymes in the tricarboxylic acid (TCA) cycle and oxygen metabolism (Neilands, 1981). While limited iron concentrations abolish bacterial growth, high intracellular iron concentrations may damage bacteria owing to the formation of undesired reactive oxygen species (ROS) (Touati, 2000; Andrews et al., 2003). In addition, bacteria must alter the expression of various virulence genes to adapt to iron availability during infection, such as those involved in siderophore production, fimbriae expression, and biofilm formation (Lin et al., 2010; Rohmer et al., 2011; Wu et al., 2012; Porcheron and Dozois, 2015). Therefore, it is important for bacteria to develop a tight regulatory circuit in response to iron availability in order to perform infection processes successfully.

*Klebsiella pneumoniae* is a gram-negative facultative anaerobe that causes community-acquired diseases including pneumonia, bacteremia, septicemia, and urinary and respiratory tract infections in patients with underlying diseases (Podschun and Ullmann, 1998). In addition, hypervirulent *K. pneumoniae* is also a major pathogen for pyogenic liver abscesses in diabetic patients (Han, 1995; Lau et al., 2000; Yang et al., 2009). Heavy capsular polysaccharide (CPS) is a characteristic of isolates causing pyogenic liver abscesses, protecting the bacteria from serum killing (Sahly et al., 2000; Lin et al., 2004). Apart from CPS, fimbriae are considered another crucial virulence factor in *K. pneumoniae* pathogenesis (Livrelli et al., 1996). Type 3 fimbriae, which are encoded by the *mrkABCDF* operon, are often expressed in heavily encapsulated *K. pneumoniae* isolates and play an important role in biofilm formation (Di Martino et al., 2003; Jagnow and Clegg, 2003; Wu et al., 2012). Biofilm formation is considered to be a key factor in the development of nosocomial infections, increasing bacterial tolerance to antibiotics, which causes problems during medical treatment (Murphy and Clegg, 2012). Therefore, a majority of *K. pneumoniae* isolates from catheter-associated urinary tract or hospital-acquired respiratory tract infections can produce functional type 3 fimbriae (Ong et al., 2010). Furthermore, the ability of *K. pneumoniae* colonization and subsequent persistence in mice was reduced when the type 3 fimbriae expression was abolished (Murphy et al., 2013). In addition, immunization of mice with purified type 3 fimbriae confers protection against following challenge with virulent *K. pneumoniae* (Lavender et al., 2005; Wang et al., 2016). Even though these findings indicate the significance of type 3 fimbriae to the virulence of *K. pneumoniae*, the regulation of the type 3 fimbrial gene expression in *K. pneumoniae* still remains largely unknown (Murphy and Clegg, 2012).

In bacteria, c-di-GMP is a bacterial second messenger that modulates biofilm formation and controls expression of the virulence genes (Tamayo et al., 2007). In *K. pneumoniae*, several studies revealed that a gene cluster adjacent to the type 3 fimbriae, *mrkHIJ*, was involved in the modulation and sensing of cyclic di-GMP (c-di-GMP) and also regulated the type 3 fimbriae expression (Wilksch et al., 2011; Murphy and Clegg, 2012; Wu et al., 2012; Yang et al., 2013). MrkH is a PilZ-domain protein that is able to bind to c-di-GMP and is a central positive regulator in type 3 fimbriae expression (Wilksch et al., 2011; Yang et al., 2013). MrkI is a LuxR-type transcriptional regulator that activates type 3 fimbriae expression (Wu et al., 2012). MrkJ possesses an EAL domain that allows it to serve as a functional c-di-GMP phosphodiesterase (PDE) for hydrolysis of c-di-GMP to further repress type 3 fimbriae expression and biofilm formation (Johnson and Clegg, 2010). Similarly, YfiN and YjcC are also c-di-GMP PDE proteins and play a negative role in control of type 3 fimbriae expression in *K. pneumoniae* (Wilksch et al., 2011; Huang et al., 2013). In addition to c-di-GMP-related proteins, several transcriptional regulators have reported to be involved in control of type 3 fimbriae expression in *K. pneumoniae*, such as histone-like nucleoid-structuring protein (H-NS), CRP, and ferric uptake regulator (Fur) (Wu et al., 2012; Ares et al., 2016; Lin et al., 2016). Thus, the regulation of type 3 fimbriae in *K. pneumoniae* in response to different environmental stimulus is more complicated than previously expected.

During the infection, iron availability is an important environmental signal affecting bacterial pathogenesis. In *K. pneumoniae*, CPS biosynthesis, type 3 fimbriae expression, and bacterial biofilm formation in *K. pneumoniae* were controlled by Fur and iron availability (Lin et al., 2010; Wu et al., 2012). Like Fur, we found that IscR also affects the expression of genes involved in the iron-acquisition system and CPS biosynthesis in *K. pneumoniae* (Wu et al., 2014). IscR acts as a crucial transcriptional regulator that controls iron–sulfur (Fe–S) cluster biosynthesis in bacteria. Fe–S clusters are important cofactors of multiple proteins involved in various cellular processes in bacteria (Andrews et al., 2003; Outten et al., 2004). Switching between the [2Fe–2S] holo and apo forms of IscR can affect the regulatory specificity of IscR to target genes in response to iron availability and oxidative stress (Outten et al., 2004; Yeo et al., 2006; Pullan et al., 2007; Giel et al., 2013). In several bacteria, IscR has been demonstrated to be implicated in pathogenesis (Wu and Outten, 2009; Lim and Choi, 2013; Miller et al., 2014; Haines et al., 2015). In *Escherichia coli*, expression of type 1 fimbriae is inhibited by apo-IscR to further reduce biofilm formation (Wu and Outten, 2009). Furthermore, IscR can induce the production of CAF/I fimbriae in response to iron depletion in enterotoxigenic *E. coli* (Haines et al., 2015). In *Vibrio vulnificus*, the *iscR* mutant reduces the expression of multiple virulence factors to further affect mouse mortality (Lim and Choi, 2013). In *Yersinia pseudotuberculosis*, IscR plays a critical role in the control of type 3 secretion and virulence (Miller et al., 2014). However, the regulatory role of IscR in *K. pneumoniae* pathogenesis remains largely unknown.

In this study, we investigated if IscR regulates type 3 fimbriae expression, biofilm formation, and virulence in *K. pneumoniae*; the role of the [2Fe–2S] cluster in IscR regulation was also evaluated. Therefore, we found that holo-IscR is able to inhibit type 3 fimbriae expression to further reduce biofilm formation. Although IscR repression of type 3 fimbriae expression occurs indirectly, we demonstrated that holo-IscR directly represses *mrkHI* expression to further reduce type 3 fimbriae expression. Furthermore, we also found that deletion of *iscR* reduces the survival rate of mice, while the *iscR*_3CA_ mutant maintains *K. pneumoniae* virulence. Taken together, our results show that IscR plays an important role in *K. pneumoniae* pathogenesis.

## Materials and Methods

### Bacterial Strains, Plasmids, and Media

All bacterial strains and plasmids used in this study are listed in **Table 1**. Primers used in this study are list in **Table 2**. Bacterial were routinely cultured at 37°C in Luria-Bertani (LB) medium supplemented with appropriate antibiotics including ampicillin (100 μg/ml), kanamycin (25 μg/ml), and streptomycin (500 μg/ml).

**Table 1:** Bacterial strains and plasmids used in this study.

Strains or plasmids	Descriptions	Reference or source
***K. pneumoniae***		
CG43S3	CG43 Sm^r^	Lai et al., 2001
Δ*iscR*	CG43S3Δ*iscR*	Wu et al., 2014
*iscR*_3CA_	CG43S3*iscR*_3CA_	Wu et al., 2014
Δ*mrkH*	CG43S3Δ*mrkH*	Wu et al., 2012
Δ*iscR-*Δ*mrkH*	CG43S3Δ*iscR*Δ*mrkH*	This study
Δ*lacZ*	CG43S3Δ*lacZ*	Lin et al., 2006
Δ*lacZ-*Δ*iscR*	CG43S3Δ*lacZ*Δ*iscR*	Wu et al., 2014
Δ*lacZ-iscR*_3CA_	CG43S3Δ*lacZiscR*_3CA_	Wu et al., 2014
Δ*lacZ-*Δ*mrkH*	CG43S3Δ*lacZ*Δ*mrkH*	Wu et al., 2012
Δ*lacZ-*Δ*iscR-*Δ*mrkH*	CG43S3Δ*lacZ*Δ*iscR*Δ*mrkH*	This study
***E. coli***		
BL21(DE3)	*F^-^ ompT hsdS_B_[r_B_^-^m_B_^-^]gal dcm* [DE3]	New England Biolabs
S17-1 *aaa pir*	*hsdR recA pro* RP4-2 [Tc::Mu; Km::Tn*7*] [*aaapir*]	Miller and Mekalanos, 1988
**Plasmids**		
pmrkAZ15	Cm^r^, 402-bp fragment containing the region upstream of *mrkA* cloned into placZ15	Lin et al., 2016
pmrkHIZ15	Cm^r^, 405-bp fragment containing the region upstream of *mrkHI* cloned into placZ15	Lin et al., 2016
pIscR	Cm^r^, 980-bp fragment containing an *iscR* allele cloned into pACYC184	Wu et al., 2014
pIscR_3CA_	Cm^r^, 980-bp fragment containing C92A, C98A and C104A mutant allele of *iscR* cloned into pACYC184	Wu et al., 2014
pET30b-IscR	Km^r^, 654-bp fragment encoding full-length IscR cloned into pET30b	Wu et al., 2014
pET30b-IscR_3CA_	Km^r^, 654-bp fragment encoding full-length C92A, C98A and C104A mutant allele of *iscR* cloned into pET30b	Wu et al., 2014

**Table 2:** Primers used in this study.

Primer	Sequence (5′→3′)	Enzyme cleaved	
GT288	CGGATCCAGACAAAATGGAGGGAACCCTA	*Bam*HI	
GT289	CAGATCTTACTGGTCTTTATCGTTCCCTC	*Bgl*II	
GT290	GCAATAGCAACATTCTGATTGG		
GT342	AATGAGAGAACGATCGTCGATCA		
GT345	TTATCCTTCGACCGGTCTCC		
GT348	AGATCCTACAAATGGGGCGT		
GT349	TCCTCAATATTTGCCTGGAA		
**For qRT-PCR**	**Sequence (5′→3′)**	**TaqMan probes**	**Target**
RT11	GGTAGGGGAGCGTTCTGTAA	67	23S rRNA
RT12	TCAGCATTCGCACTTCTGAT		
RT29	TAAGCAAACTGGGCGTGAA	20	*mrkA*
RT30	TAGCCCTGTTGTTTGCTGGT		
GT46	GTTTAAGTTCCGCCATCTCG	120	*mrkH*
GT47	TTGCGCTTGGCTTCTAAGAT		
GT42	AGTTATGCCGATGTCATCCAT	59	*mrkI*
GT43	GATTCTGATGGCAGAAATATCCTT		
GT54	TTTCGAGGTAACCGAAAACG	84	*mrkJ*
GT55	GAGGTATCCTGTGGGCTCTG		

### Western Blotting

The total proteins of exponential phase *K. pneumoniae* cultures were separated by SDS-PAGE (approximately 5 μg per lane) and transferred to PVDF membrane. Western analysis was followed as previously described (Lin et al., 2016). Rabbit anti-MrkA antibody was used as the primary antibody. Goat anti-rabbit immunoglobulin G antibody conjugated to horseradish peroxidase (Abcam) was used as the secondary antibody. After incubation with corresponding antibody, the signal in the membranes was collected by ImageQuant LAS 4000 mini (GE Health, United States) after the visualization with an enhanced chemiluminescence ECL western blotting luminal reagent (PerkinElmer, Wellesley, MA, United States).

### Quantitative Reverse-Transcription PCR (qRT-PCR)

Total RNA extraction, reverse transcription of isolated mRNA to cDNA, qRT-PCR, and data analysis were performed according to the previous study (Lin et al., 2013). Primers and probes were designed for selected target sequences using Universal ProbeLibrary Assay Design Center (Roche-applied science) and shown in **Table 2**. Relative gene expressions were quantified using the comparative threshold cycle 2^-ΔΔC_T_^ method with 23S rRNA as the endogenous reference.

### Measurement of Promoter Activity

To generate the promoter region of *mrkHI* that lacked #2 type 1 IscR box, the plasmid, pmrkHIZ15, was used as the template for the inverse-PCR with the primer pair GT342/GT345 to generate the DNA fragment of P*_mrkHIΔ2_*. Subsequently, the purified DNA fragment was treated with *Dpn*I for 2 h, and then subject to T4 PNK treatment and self-ligation. The ligation product was transformed into *E. coli* DH5α and confirmed by DNA sequencing. Then, the DNA fragment containing P*_mrkHIΔ2_* was subcloned into placZ15 to generate pmrkHIZ15Δ2. The DNA fragment of P*_mrkHIΔ1_*, which lacked #1 type 1 IscR box, was also generated by PCR-amplified with primer pair GT348/GT289 and the amplicon was then cloned into placZ15 to generate pmrkHIZ15Δ1. Finally, the promoter-reporter plasmids, pmrkAZ15, pmrkHIZ15, pmrkHIZ15Δ2, and pmrkHIZ15Δ1, were mobilized into *K. pneumoniae* strains by electroporation, respectively. The β-galactosidase activity of logarithmic phase bacteria was measured as previously described (Lin et al., 2006).

### Biofilm Formation

Biofilm formation was assessed by the ability of the cells to adhere to the walls of 96-well microtitre dishes made of PVC (TPP 96 flat) with some modification of the reported protocol (Lembke et al., 2006). The plate contained an aliquot of 1:10 diluted overnight bacteria culture and then was incubated at 37°C statically for 24 h for biofilm formation. The un-adherent bacteria was washed triply with 200 μl PBS and then adherent bacteria was stained with 200 μl of 0.1% safranin solution at room temperature for 30 min. The plates was rinsed twice with deionised water to remove excess stain. Finally, the safranin stained biofilm was solubilized in 200 μl of 95% ethanol and the absorbance determined at a wavelength of 492 nm.

### Purification of IscR::His_6_ and IscR_3CA_::His_6_

The plasmids, pET30b-IscR and pET30b-IscR_3CA_, in *E. coli* BL21(DE3)[pLysS] (Invitrogen, United States) was used to overexpress the recombinant proteins IscR::His_6_ and IscR_3CA_::His_6_, respectively. The detail of expression and purification of the recombinant proteins was followed as previously described (Wu et al., 2014).

### Electrophoretic Mobility Shift Assay (EMSA)

DNA fragments of the putative promoter region of *mrkHI* were amplified with Pfu polymerase using the indicated primer sets to generate DNA probes for EMSA (*P*_mrkHI-1_ and *P*_mrkHI-3_). To obtain DNA fragment of *P*_mrkHI-2_, the plasmid pmrkHIZ15Δ2 was as a template to be amplified with GT349/GT290 for DNA probe in EMSA. EMSA was performed as previously described (Wu et al., 2014). The assay was repeated in at least 3 independent experiments.

### The Peritonitis Model of Mouse Infection

To evaluate the role of *iscR* in *K. pneumoniae* virulence, the 8-weeks male BALB/c mice (National Laboratory Animal Center, Taiwan) were injected intraperitoneally with 100 μl of bacterial suspension containing 5 × 10^4^ CFU of mid-log *K. pneumoniae* strains. The survival rate of the infected mice was monitored daily for 10 days.

### Ethics Statement

All the animal experiments were followed as the recommendation in the Guide for the Care and Use of Laboratory Animals of the National Laboratory Animal Center (NLAC, Taiwan). The animal protocols were approved by China Medical University Experimental Animal Center (Permit number: 2016-212).

### Statistical Method

The results of qRT-PCR analysis and promoter activity were performed at least triplicate. The results are showed as the mean and standard deviation. Differences between groups were evaluated by an unpaired *t*-test. The survival rate was determined by log-rank test using GraphPad Prism 5.0. Values of *P* < 0.05 and *P* < 0.01 were considered statistically significant difference.

## Results

### Effect of the State of the Fe-S Cluster in IscR on Type 3 Fimbriae Expression

**FIGURE 1:**
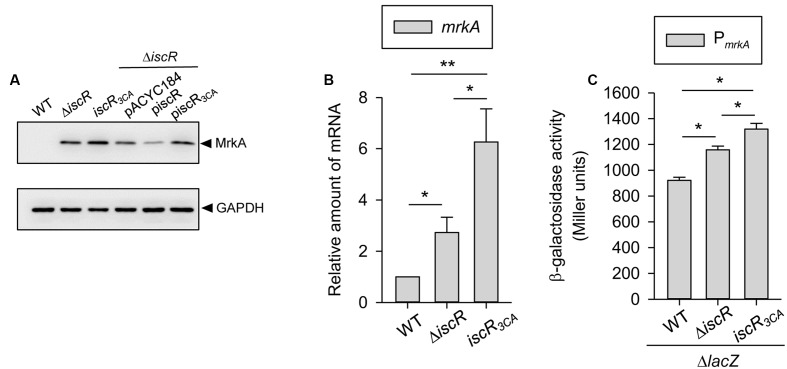
IscR affects the type 3 fimbriae expression in *K. pneumoniae*. *K. pneumoniae* CG43S3 WT, Δ*iscR*, and *iscR*_3CA_ strains was grown to mid-log phase at 37°C in LB broth to observe the type 3 fimbriae expression by **(A)** western blot analysis against MrkA (the upper panel) and GAPDH antiserum (the lower panel, for internal control). The MrkA and GAPDH proteins are indicated by an arrow, respectively, and **(B)** qRT-PCR analyses of *mrkA* gene expression. **(C)** β-galactosidase activities of *K. pneumoniae* CG43S3 Δ*lacZ* and the isogenic strains (Δ*lacZ-*Δ*iscR* and Δ*lacZ-iscR*_3CA_) carrying the reporter plasmid pmrkAZ15 (P*_mrkA_*::*lacZ*) were determined using log-phase cultures grown in LB medium. The results are representative of three independent experiments. Error bars indicate standard deviations. ^∗^*P* < 0.05 and ^∗∗^*P* < 0.01 compared to the indicated group.

To study whether IscR regulates *K. pneumoniae* type 3 fimbriae expression, we determined the levels of MrkA (the major subunit of type 3 fimbriae) in CG43S3 (WT) and Δ*iscR* strains. Compared with the levels in the WT, Δ*iscR* produced higher amounts of MrkA (**Figure 1A**), suggesting that IscR represses type 3 fimbriae expression. To further investigate the role of the [2Fe–2S] cluster in the IscR regulation of type 3 fimbriae, an *iscR* mutant (named *iscR*_3CA_), created by replacing three cysteines with alanines to mimic apo-IscR, was used to observe type 3 fimbriae expression. Likewise, we also found that levels of MrkA were higher in the *iscR*_3CA_ strain than in the WT strain, suggesting that the [2Fe–2S] cluster is required for the repression of type 3 fimbriae expression by IscR. However, we noted that higher levels of MrkA production were found in the *iscR*_3CA_ strain than in the Δ*iscR* mutant, implying that apo-IscR acts as an activator in controlling type 3 fimbriae expression. To further confirm the role of the [2Fe–2S] cluster in IscR regulation of fimbriae expression, the empty vector (pACYC184) and complement plasmids pIscR and pIscR_3CA_ were introduced into the Δ*iscR* strain to observe MrkA production. Compared to the MrkA expression in Δ*iscR* [pACYC184], the introduction of the complement plasmid pIscR into Δ*iscR* repressed the MrkA production level, whereas a slight increase in MrkA production was found in Δ*iscR* [pIscR_3CA_]. These results suggest that IscR plays a dual role in the regulation of type 3 fimbriae expression and that the status of the [2Fe–2S] cluster in IscR is critical for this regulation. Furthermore, to further study the activity of IscR as a transcriptional regulator in control of type 3 fimbriae expression, mRNA levels of *mrkA* were measured in the WT, Δ*iscR*, and *iscR*_3CA_ strains by qRT-PCR. As shown in **Figure 1B**, the mRNA levels of *mrkA* were higher in the Δ*iscR* and *iscR*_3CA_ strains than in the WT strain. In addition, the mRNA levels of *mrkA* in the *iscR*_3CA_ strain were higher than those in the Δ*iscR* strain. Furthermore, to further identify IscR could affect the promoter activity of *mrkA*, a plasmid carrying P*_mrkA_* fused with the *lacZ* reporter gene was constructed and introduced into the Δ*lacZ*, Δ*lacZ-*Δ*iscR*, and Δ*lacZ-iscR*_3CA_ strains, respectively. As shown in **Figure 1C**, P*_mrkA_* activity was higher in the Δ*lacZ-*Δ*iscR* strain than in the Δ*lacZ* strain. In addition, P*_mrkA_* activity was significantly higher in the Δ*lacZ-iscR*_3CA_ strain than in the Δ*lacZ-*Δ*iscR* strain. Taken together, this confirms that the status of the [2Fe–2S] cluster in IscR is critical for regulating type 3 fimbriae expression at the transcriptional level.

### IscR Represses Biofilm Formation

**FIGURE 2:**
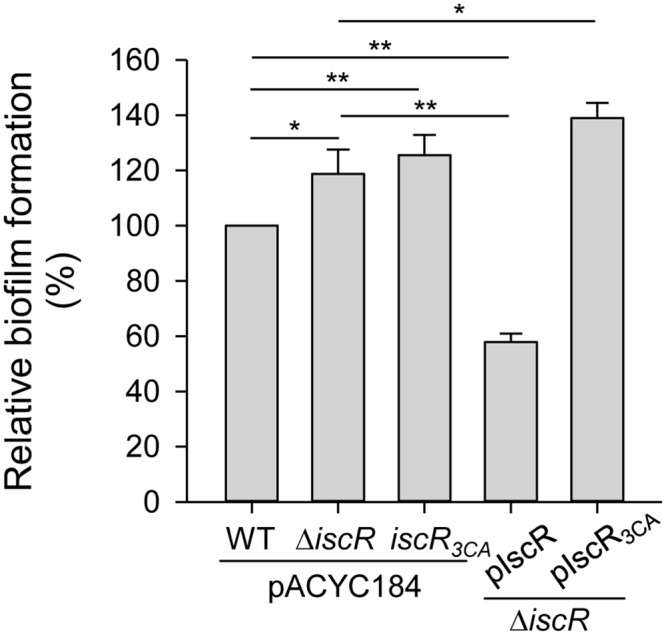
Effect of IscR on biofilm formation. *K. pneumoniae* strains, WT [pACYC184], Δ*iscR* [pACYC184], *iscR*_3CA_ [pACYC184], Δ*iscR* [piscR], and Δ*iscR* [piscR_3CA_], were grown at 37°C for 24 h in LB, and bacterial biofilm formation was quantified as described in “Materials and Methods.” The results are representative of three independent experiments. Error bars indicate standard deviations. ^∗^*P* < 0.05 and ^∗∗^*P* < 0.01 compared to the indicated group.

Based on the fact that type 3 fimbriae are a major mediator of biofilm formation, we further speculated that IscR could affect *K. pneumoniae* biofilm formation. As shown in **Figure 2**, we found that the Δ*iscR* [pACYC184] strain resulted in a slight increase in biofilm formation compared with that in theWT [pACYC184] strain, confirming that the repression of type 3 fimbriae activity by IscR was also reflected in biofilm formation. To further investigate the role of the [2Fe–2S] cluster in the IscR regulation of type 3 fimbriae, *iscR*_3CA_ [pACYC184] was used to observe biofilm formation. Biofilm formation activity was elevated in the *iscR*_3CA_ strain compared with that in the WT [pACYC184] strain. This indicates that the [2Fe–2S] cluster of IscR is required for the repression of biofilm formation. Furthermore, the introduction of the complement plasmid pIscR into the Δ*iscR* strain repressed biofilm formation as compared with that in Δ*iscR* [pACYC184] and Δ*iscR* [pIscR_3CA_], confirming that holo-IscR acts as a negative regulator in controlling biofilm formation. In addition, we noted that biofilm formation was stronger in Δ*iscR* [pIscR_3CA_] than in Δ*iscR* [pACYC184], implying that apo-IscR plays a positive role in biofilm formation.

### No Binding Activity of IscR in the Promoter of mrkA

For further investigation of the mechanism of IscR regulation of *mrkA* transcription, sequences of putative IscR binding sites were manually analyzed in the promoter region of *mrkA*. We found a putative type 1 IscR box (5′-ATAACTTAATGAAACGTGAACAAAT-3′) with 60% (15/25 bp) homology to the consensus sequence of *E. coli* located between -15 and -39 bp relative to the transcriptional start codon of *mrkABCDF* (type 3 fimbriae gene cluster). Therefore, an electrophoretic mobility shift assay (EMSA) was performed to demonstrate whether IscR could directly bind to the promoter region of *mrkABCDF*. We found that the purified recombinant IscR::His_6_ protein was unable to bind to the promoter region of *mrkABCDF* (data not shown). In addition, we also noted a putative type 2 IscR box (5′-ACCACCCTCGCGTTTTCATCTATCAA-3′) with about 69% (18/26 bp) homology to the consensus sequence of *E. coli* located between -75 and –100 bp relative to the transcriptional start codon of *mrkABCDF.* However, the purified recombinant IscR_3CA_::His_6_ protein did not bind to this sequence in the promoter region of *mrkABCDF* either (data not shown). Therefore, these results indicate that IscR represses type 3 fimbriae expression indirectly.

### MrkH and MrkI Are Involved in IscR Regulation of Type 3 Fimbriae

**FIGURE 3:**
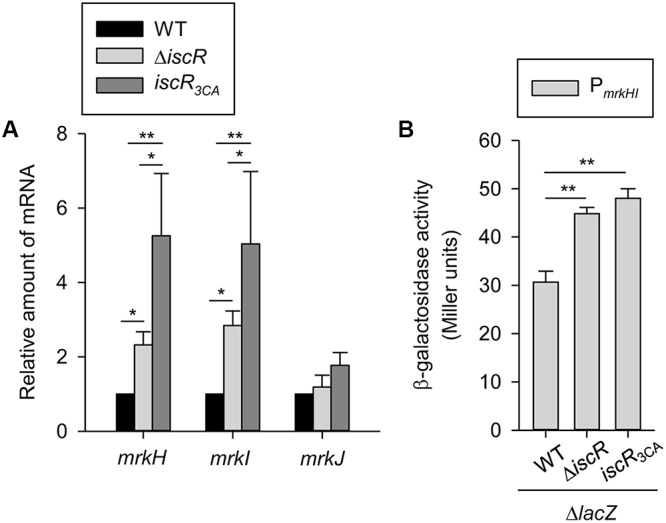
Effect of IscR on *mrkH*, *mrkI*, and *mrkJ* expression. **(A)** qRT-PCR analyses of the *mrkH*, *mrkI*, and *mrkJ* expressions for WT, Δ*iscR*, and *iscR*_3CA_ strains in LB medium. **(B)** β-galactosidase activities of *K. pneumoniae* CG43S3 Δ*lacZ* and the isogenic strains (Δ*lacZ-*Δ*iscR* and Δ*lacZ-iscR*_3CA_) carrying the reporter plasmid pmrkHZ15 (P*_mrkHI_*::*lacZ*) were determined using log-phase cultures grown in LB medium. The results are representative of three independent experiments. Error bars indicate standard deviations. ^∗^*P* < 0.05 and ^∗∗^*P* < 0.01 compared to the indicated group.

A c-di-GMP-related gene cluster (*mrkHIJ*) adjacent to the type 3 fimbriae operon has been demonstrated to regulate type 3 fimbriae expression (Murphy and Clegg, 2012; Wu et al., 2012). To further investigate whether *mrkHIJ* are involved in the IscR regulon, the effect of *iscR* deletion on the mRNA levels of these genes was determined by qRT-PCR. Compared with levels in the WT strain, the mRNA levels of *mrkH* and *mrkI* were markedly increased in the Δ*iscR* and *iscR*_3CA_ strains, while no apparent effect was found on the mRNA level of *mrkJ* (**Figure 3A**). This indicates that the [2Fe-2S] cluster of IscR is required for repressing the expression of *mrkH* and *mrkI*. Furthermore, we also noted that the expression levels of *mrkH* and *mrkI* in the *iscR*_3CA_ strain were higher than those in the Δ*iscR* strain. This implies that apo-IscR activates *mrkH* and *mrkI* expression. Likewise, the activities of P*_mrkHI_* were also higher in the Δ*lacZ-*Δ*iscR* and Δ*lacZ-iscR*_3CA_ strains than in the Δ*lacZ* strain (**Figure 3B**), but no apparent difference was found between the Δ*lacZ-*Δ*iscR* and Δ*lacZ-iscR*_3CA_ strains, indicating that holo-IscR acts a transcriptional repressor of *mrkHI* expression.

### IscR Directly Binds the Promoter of mrkHI

**FIGURE 4:**
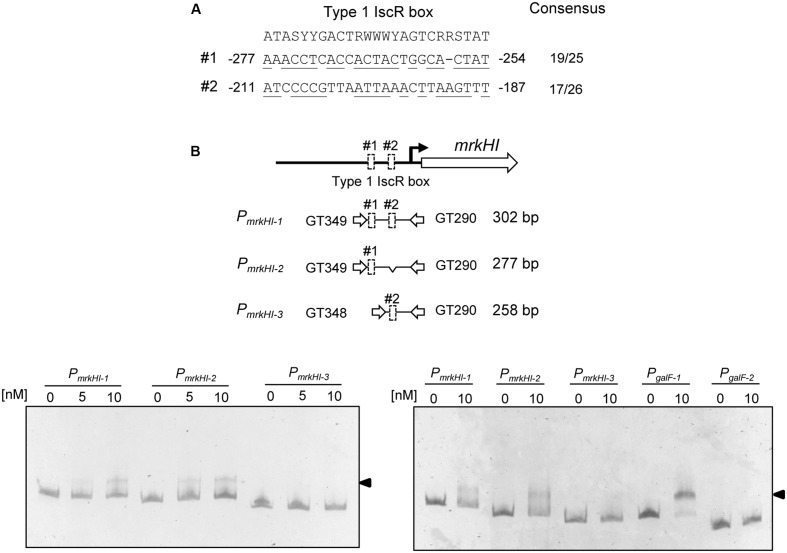
IscR represses directly the *mrkHI* expression. **(A)** DNA sequence alignment between the *E. coli* type 1 IscR box and the putative IscR binding sequence in the upstream region of *mrkH*. Positions identical to the consensus sequences are underlined. **(B)** Diagrammatic representation of the upstream of *mrkH* (the upper panel). The large arrows represent the open reading frames. The primer sets used in PCR amplification of the DNA probes are indicated, and the numbers denote the DNA amplified length. The predicted type 1 IscR boxes are deleted and indicated by an open box. Different concentrations of purified IscR::His6 were incubated with 5 ng of various DNA fragments of the upstream regions of *mrkH*. Following incubation at room temperature for 30 min, the mixtures were analyzed on a 5% non-denaturing polyacrylamide gel. The gel was stained with SYBR Green I dye and photographed.

For further investigation of the mechanism of IscR regulation of *mrkHI* transcription, sequences of putative IscR binding sites were manually analyzed in the promoter region of *mrkHI*. As shown in **Figure 4A**, we found two predicted type 1 IscR boxes (#1 and #2) located at -277 to -254 and -211 to -187 relative to the translation start site of *mrkHI*, respectively. The #1 and #2 sites are 76% (19/25 bp) and 68% (17/25 bp) homologous, respectively, with the consensus sequence of *E. coli*. We hypothesized that IscR would bind directly to the #1 or #2 site in the promoter region of *mrkHI* to repress gene transcription, and we confirmed this by performing an EMSA. As shown in **Figure 4B**, we found that IscR::His_6_ could directly bind to *PmrkHI-1*, *PmrkHI-2*, and *PgalF-1* (as a positive control), whereas no specific binding between IscR::His_6_, and *PmrkH-3* or *PgalF-2* (as a negative control) was found. Although the binding activity of IscR::His_6_ to *PmrkHI-1* and *PmrkHI-2* seemed to be weaker than that to *PgalF-1*, the intensity of the shifted bands slightly increased when the protein concentration was increased (**Figure 4B**). This result supports that IscR could specifically bind to the #1 site in the promoter region of *mrkHI* to control *mrkHI* expression. In addition, compared with that of IscR::His_6_, the recombinant [2Fe–2S]-clusterless IscR, IscR_3CA_::His_6_, was unable to bind *PmrkHI-1* (Data not shown). These results indicate a direct interaction between IscR and the #1 site of the type 1 IscR box in the *mrkHI* promoter and confirm that the [2Fe–2S] cluster of IscR plays a crucial role in this interaction.

### IscR Regulates the Expression of Type 3 Fimbriae through MrkH and an Unknown Factor

**FIGURE 5:**
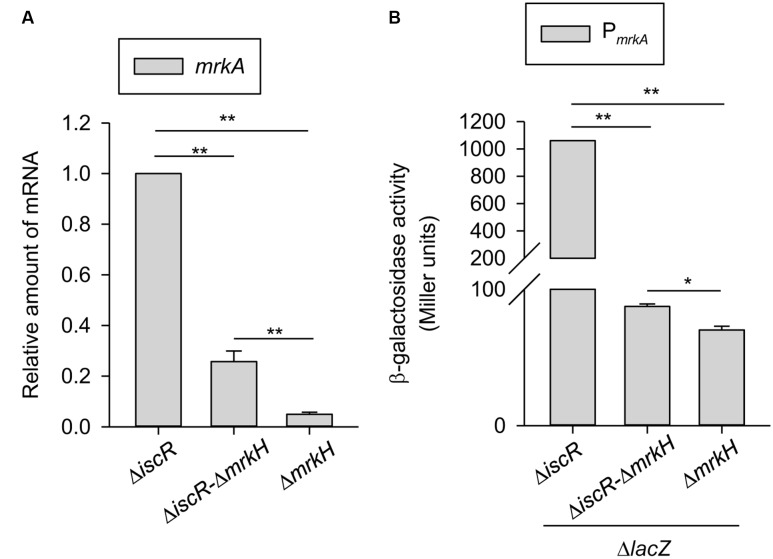
Role of MrkH in IscR regulation of type 3 fimbriae expression. **(A)** qRT-PCR analyses of *mrkA* expression in Δ*iscR*, Δ*iscR-*Δ*mrkH*, and Δ*mrkH* strains which was grown in LB medium. **(B)** β-galactosidase activities of *K. pneumoniae* CG43S3 Δ*lacZ-*Δ*iscR*, Δ*lacZ-*Δ*iscR-*Δ*mrkH*, and Δ*lacZ-*Δ*mrkH* carrying the reporter plasmid pmrkAZ15 (P*_mrkA_*::*lacZ*) were determined using log-phase cultures grown in LB medium. The results are representative of three independent experiments. Error bars indicate standard deviations. ^∗^*P* < 0.05 and ^∗∗^*P* < 0.01 compared to the indicated group.

Although MrkH is a well-known regulator of type 3 fimbriae (Wilksch et al., 2011; Yang et al., 2013), we wanted to identify whether MrkH is the sole transcriptional regulator in the regulation of type 3 fimbriae expression by IscR. An Δ*iscR*Δ*mrkH* strain was generated, and the mRNA expression of *mrkA* was analyzed in the Δ*iscR*, Δ*iscR*Δ*mrkH*, and Δ*mrkH* strains. As shown in **Figure 5A**, the mRNA expression of *mrkA* in the Δ*iscR*Δ*mrkH* strain was significantly higher than that in the Δ*mrkH* strain. Similarly, the promoter activity of *mrkA* also confirmed this finding; although the effect was weak, it was still significant (**Figure 5B**). This suggests that in addition to MrkH, another transcriptional factor is also involved in the repression of type 3 fimbriae expression by IscR.

### Role of IscR in *K. pneumoniae* Virulence

**FIGURE 6:**
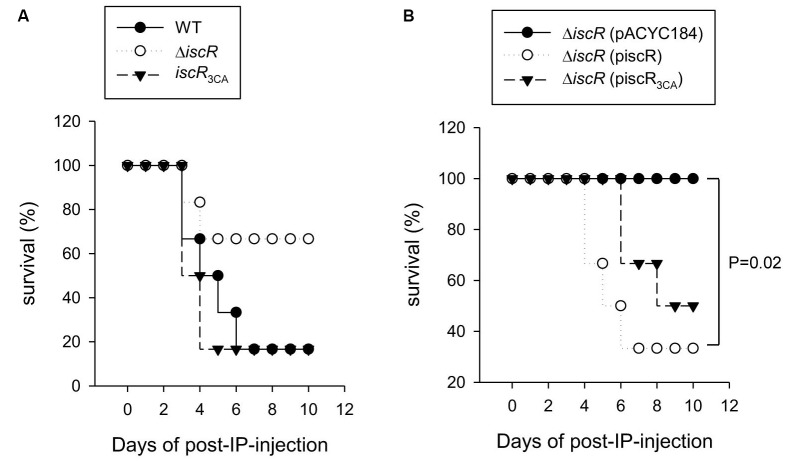
Analysis of *iscR* on *K. pneumoniae* virulence to survival rate of mice. The survival rate of *K. pneumoniae* CG43S3 **(A)** WT (dark spot), Δ*iscR* (open spot), or *iscR*_3CA_ (dark triangle) and **(B)** the complement strains, Δ*iscR* [pACYC184] (dark spot), Δ*iscR* [pIscR] (open spot), or Δ*iscR* [pIscR_3CA_] (dark triangle) infected mice (*n* = 6 per group) was monitored daily for 10 days and determined by log-rank test using GraphPad Prism 5.0; *P*-value of <0.05 was considered statistically significant.

To understand the effect of the deletion of *iscR* on *K. pneumoniae* virulence, the survival of mice that were intraperitoneally inoculated with the WT, Δ*iscR*, or *iscR*_3CA_ strains was monitored for 10 days. As shown in **Figure 6A**, when mice were inoculated with the WT, Δ*iscR*, and *iscR*_3CA_ strains, survival decreased to 16.7, 66.7, and 16.7% by day 10, respectively. The deletion of *iscR* showed a trend to attenuate the bacterial virulence; however, the difference did not reach statistical significance. In addition, this result also implied that the absence of the [2Fe–2S] cluster of IscR did not eliminate *K. pneumoniae* virulence during infection. To further confirm the result, the mice were intraperitoneally inoculated with Δ*iscR* [pACYC184], Δ*iscR* [pIscR], and Δ*iscR* [pIscR_3CA_] to monitor the survival rate. As shown in **Figure 6B**, we found that mice inoculated with Δ*iscR* [pACYC184], Δ*iscR* [pIscR], or Δ*iscR* [pIscR_3CA_] exhibited survivals of 100, 33.3, and 50% by day 10, respectively. The complementation of *iscR* significantly decreased the survival of mice, suggesting that IscR is involved in the *K. pneumoniae* virulence. Besides, the complementation of iscR_3CA_ appeared to partly restore the bacterial virulence; however, the difference is not statistically different.

## Discussion

In many bacteria, iron-responsive regulators play important roles in controlling the expression of several virulence factors according to iron availability (Wu and Outten, 2009; Wu et al., 2012; Lim and Choi, 2013; Miller et al., 2014). Previously, we demonstrated that iron availability affects CPS, iron acquisition systems, type 3 fimbriae expression, and biofilm formation in *K. pneumoniae* (Lin et al., 2010; Wu et al., 2012, 2014). In addition, while the regulatory role of IscR in CPS biosynthesis and the iron-acquisition system have been demonstrated (Wu et al., 2014), the role of IscR in *K. pneumoniae* pathogenesis had not been entirely elucidate. In this study, we found that type 3 fimbriae expression and biofilm formation were also affected by the status of [2Fe–2S] cluster of IscR, playing a critical role in mediating the expression of virulence factors for successful infection.

In *K. pneumoniae*, Fur has been demonstrated to play a central role in directly activating *mrkA* and *mrkH* expression to increase biofilm formation (Wu et al., 2012). In this study, we found that, compared to the WT strain, both the *K. pneumoniae* Δ*iscR* and *iscR*_3CA_ strains harbored increased the type 3 fimbriae expression and biofilm-forming activities, which could be reversed by the complementation of pIscR, but not pIscR_3CA_ into the Δ*iscR* strain (**Figures 1**, **2**). Likewise, overexpression of pIscR in *K. pneumoniae* CG43S3 also repressed the mRNA expression of *mrkA*, while pIscR_3CA_ in *K. pneumoniae* CG43S3 activated the *mrkA* expression (Supplementary Figure S1). These results suggest that IscR represses type 3 fimbriae expression and biofilm formation in a Fe–S-cluster-dependent manner. Although Fur and IscR exert positive and negative regulatory effects, respectively, on *K. pneumoniae* biofilm formation under iron-repleted conditions, we speculated that Fur played a major role, since *iscR* expression is repressed in response to environmental iron (Wu et al., 2014). Furthermore, although type 3 fimbriae are critical in *K. pneumoniae* biofilm formation, it has also been demonstrated that the function of fimbriae is hindered by the concomitant expression of a thick capsule on the bacterial surface (Schembri et al., 2005; Goncalves Mdos et al., 2014). Previously, we found that holo-IscR but not apo-IscR could activate CPS production (Wu et al., 2014). In **Figure 1**, the repressive effect of IscR on MrkA production at the translational level was more apparent than that at the transcriptional level. We hypothesized that the reduced CPS production in the Δ*iscR* strain would promote the assembly of type 3 fimbriae on the bacterial surface. Moreover, we also found that type 3 fimbriae expression is slightly increased in the *iscR*_3CA_ strain as compared to that in the WT strain (**Figure 1**). In contrast to pIscR, the introduction of pIscR_3CA_ into the Δ*iscR* strain increased *K. pneumoniae* biofilm formation (**Figure 2B**). It implies that apo-IscR acts as an activator to increase biofilm formation. However, the recombinant protein IscR_3CA_::His_6_ did not bind to the promoter regions of *mrkA* or *mrkH*, suggesting that apo-IscR indirectly regulates the expression of type 3 fimbriae. In *K. pneumoniae*, c-di-GMP is a critical second messenger that influences biofilm formation and type 3 fimbriae expression (Johnson and Clegg, 2010; Wilksch et al., 2011; Wu et al., 2012). To identify possible IscR-regulated genes that are involve in c-di-GMP signaling, we analyzed the upstream sequences of c-di-GMP-related genes in *K. pneumoniae* CG43, (Lin et al., 2016). We found a typical type 2 IscR box exhibiting more than 73% homology with the *E. coli* consensus sequence upstream of the c-di-GMP-related genes D364_06025 and D364_22720 in *K. pneumoniae* CG43 (data not shown). Apo-IscR may affect biofilm formation and type 3 fimbriae expression via the regulation of c-di-GMP-related gene expression, which awaits further investigation.

Holo-IscR directly repressed *mrkHI* expression to affect type 3 fimbriae expression, while apo-IscR appeared to indirectly activate *mrkHI* expression (**Figures 3**, **4**). In many bacteria, small non-coding RNAs play a critical role in post-transcriptional regulation, allowing bacteria to adapt to various environmental stimuli (Waters and Storz, 2009). RyhB is a well-known iron-responsive small RNA in bacteria (Oglesby-Sherrouse and Murphy, 2013), and we previously demonstrated that apo-IscR can directly activate several iron uptake systems that affect intracellular iron homeostasis (Wu et al., 2014). In *K. pneumoniae*, RyhB is involved in the regulation of CPS biosynthesis and the iron-acquisition system (Huang et al., 2012). Because small RNA regulates its target mRNAs via base pairing (Masse et al., 2003; Geissmann and Touati, 2004), the 5′ untranslated region (5′ UTR) of *mrkHI* mRNA was analyzed for sequences complementary to the RyhB sequence. No apparent potential interacting site was found by using RNAhybrid in BiBiServ (Rehmsmeier et al., 2004). Therefore, whether other small RNAs are involved in IscR regulation of type 3 fimbriae expression requires further investigation. Recently, H-NS was also reported as an activator in control of type 3 fimbriae expression and biofilm formation in *K. pneumoniae* (Ares et al., 2016). Deletion of *hns* increased the mRNA expression of *mrkH*, *mrkI*, and *mrkJ*. Thus, H-NS may affect the c-di-GMP concentration, through activation of *mrkJ* expression, to further influence MrkH and MrkI activity and type 3 fimbriae expression (Ares et al., 2016). As shown in **Figure 3A**, deletion of *iscR* increased the mRNA levels of *mrkH* and *mrkI* but not *mrkJ*; thus, IscR and H-NS seemed to regulate the expression of type 3 fimbriae in different manners.

To further analyze the DNA sequence of P*_mrkH_* to observe whether other transcriptional regulator is also involved in *mrkH* regulation, we found a putative binding site of phosphorylated ArcA is overlapped with the #2 site that displays 80% (12/15 bp) homology with the consensus sequence of *E. coli* (Liu and De Wulf, 2004). ArcA acts as the transcriptional regulator in the ArcA/B two-component system, regulating gene expression to adapt to aerobic and microaerobic conditions (Iuchi and Weiner, 1996; Alexeeva et al., 2003). Oxygen-limited conditions may trigger the kinase activity of ArcB, which then phosphorylates and activates the DNA binding activity of ArcA (Carpenter and Payne, 2014). Therefore, we suggest that the expression of *mrkH* and type 3 fimbriae could be affected by phosphorylated ArcA in response to redox growth conditions. This possibility should be further investigated. Furthermore, a slightly higher expression level of *mrkA* was found in Δ*iscR*Δ*mrkH* as compared with that in Δ*mrkH* (**Figure 5**). This indicates that IscR may affect other transcriptional regulator(s) besides MrkH in order to influence type 3 fimbriae expression. In addition to MrkH, MrkI is a LuxR-type transcriptional regulator containing a conserved aspartate residue (D56) able to receive a phosphorylated group for affecting its regulatory activity. In our previous study, we have demonstrated that a D56A site-directed MrkI mutant, which loss the phosphorylated state in MrkI, exhibited to decrease MrkA production as compared to that in a D56E site-directed MrkI mutant, which mimic the phosphorylate state of MrkI (Wu et al., 2012). Therefore, whether IscR could also affect the phosphorylation of MrkI to further influence type 3 fimbriae expression remains to be studied.

During infection, Fe–S cluster homeostasis in bacteria is deeply affected by iron starvation and oxidative stress conditions (Miller and Britigan, 1997; Wilks and Burkhard, 2007; Py et al., 2011). These stimuli may affect the functions of IscR in controlling gene expression during infection. In **Figure 6A**, we found that IscR is required for regulating the virulence of *K. pneumoniae* during infection in a mouse peritonitis model. Furthermore, the *iscR*_3CA_ mutant exhibited a similar virulence to that of the WT strain. In *K. pneumoniae*, CPS and iron-acquisition systems are critical and important virulence factors for successful infection (Lin et al., 2004; Regueiro et al., 2006; Russo et al., 2015). We have previously demonstrated that CPS can be activated by holo-IscR, while the three iron-acquisition systems (*fhuA*, *sitA*, and *iucA*) are directly activated by apo-IscR (Wu et al., 2014). It may be described that the deletion of *iscR* in *K. pneumoniae* reduces CPS production and iron-acquisition system expression to further diminish virulence during infection, while the introduction of pIscR into the Δ*iscR* strain increases virulence compared with that of the vector alone or pIscR_3CA_ in the Δ*iscR* strain. Furthermore, aerobactin (*iuc*) plays a critical role in the growth and survival of highly virulent *K. pneumoniae* strains (Russo et al., 2015). It may be that high expression of aerobactin biosynthesis is one of the factors used by the *iscR*_3CA_ mutant for maintaining a similar virulence to that of the WT strain. In addition, overexpression of *suf*, which is another Fe-S assembly gene cluster, in the *iscR*_3CA_ mutant leads to a defect in the proton motive force in *E. coli* and *Y. pseudotuberculosis* (Ezraty et al., 2013; Miller et al., 2014). Such a defect could influence the expression of multiple virulence factors, such as antibiotic resistance, the type 3 secretion pathway, and bacterial mobility (Ezraty et al., 2013; Miller et al., 2014). In *K. pneumoniae* CG43S3, we also found that the mRNA level of *sufA* was apparently increased in the *iscR*_3CA_ mutant as compared to that in the WT and Δ*iscR* strains (Supplementary Figure S2A). This means that apo-IscR could activate the *suf* operon, as it does in *E. coli* and *Y. pseudotuberculosis*. However, compared to the WT strain, no apparent effect on growth rate was found in the Δ*iscR* and *iscR*_3CA_ strains grown in LB medium (Supplementary Figure S2B). Therefore, whether overexpression of *suf* in the *iscR*_3CA_ mutant affects the proton motive force in *K. pneumoniae* remains to be investigated.

Aside from the role of the CPS and iron-acquisition systems during infection, type 3 fimbriae play crucial roles in adhesion to host cells, persistence, and biofilm formation (Hornick et al., 1992; Tarkkanen et al., 1997; Sebghati and Clegg, 1999; Jagnow and Clegg, 2003). Furthermore, type 3 fimbriae are key factors that affect the ability of *K. pneumoniae* to colonize and subsequently persist in mice (Murphy et al., 2013). Therefore, we suggest that IscR is also involved in *K. pneumoniae* colonization and adherence with eukaryotic cells and catheters through the regulation on the type 3 fimbriae expression and the bacterial biofilm forming activity. The role of IscR in *K. pneumoniae* virulence according to different infectious routes and host interactions needs to be further evaluated. In addition, IscR also plays an important role in mediating proper levels of Fe–S cluster biosynthesis in bacteria via transcriptional repression of the *iscRSUA* operon (Yeo et al., 2006; Giel et al., 2013), indicating that the manner in which IscR impacts *K. pneumoniae* virulence is dependent on the conditions that *K. pneumoniae* encounters during the course of infection.

Taken together, our results show that IscR can directly repress *mrkHI* expression to affect type 3 fimbriae expression and biofilm formation. Furthermore, the deletion of *iscR* decreases *K. pneumoniae* virulence during infection, demonstrating that IscR is implicated in *K. pneumoniae* pathogenesis.

## Author Contributions

Conceived and designed the experiments: T-HL, CY-T, and C-TL. Performed the experiments: T-HL, Y-CL, C-CW, and C-TL. Analyzed the data: C-YT, Y-CL, and CL. Contributed reagents/materials/analysis tools: C-YT, Y-CL, C-CW, and C-FH. Wrote the paper: T-HL and C-TL. All authors read and approved the final manuscript.

## Conflict of Interest Statement

The authors declare that the research was conducted in the absence of any commercial or financial relationships that could be construed as a potential conflict of interest.
