# Supplementary material for: IscR Regulation of Type 3 Fimbriae Expression in Klebsiella pneumoniae CG43
Source: Front Microbiol. 2017 Oct 16;8:1984. doi: 10.3389/fmicb.2017.01984 (PMC5650617; doi:10.3389/fmicb.2017.01984)
Supplement: Supplementary file 1 [file Image_1.PDF]

Supplementary Materials:

IscR Regulation of Type 3 Fimbriae Expression in *Klebsiella pneumoniae* CG43

Tien-Huang Lin, Cheng-Yin Tseng, Yi-Chyi Lai, Chien-Chen Wu, Chun-Fa Huang, Ching-Ting Lin*

*** Correspondence:**
Ching-Ting Lin
gingting@mail.cmu.edu.tw

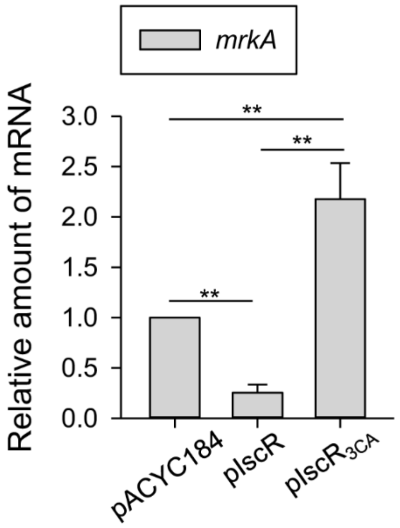

Figure S1. Expression of type 3 fimbriae in *K. pneumoniae* CG43S3 carrying pACYC184, pIscR, and pIscR3$_{CA}$.

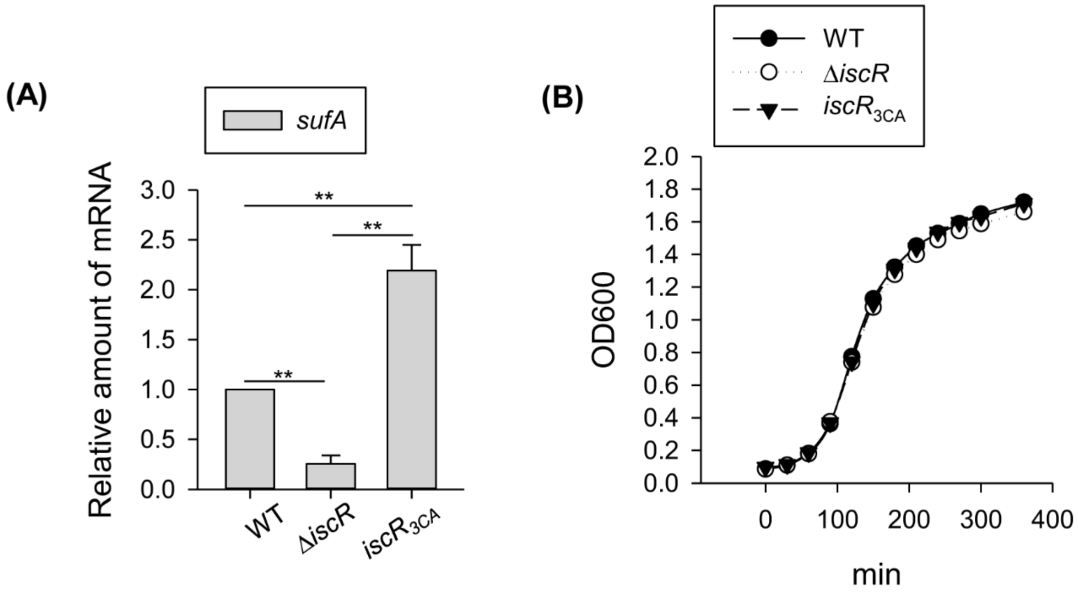

Figure S2. (A) The mRNA expression of *sufA* and (B) growth curve in *K. pneumoniae* CG43S3 WT, Δ*iscR*, and *iscR*3CA strains when bacteria grown in LB medium.
